# Real-time human progress estimation with online dynamic time warping for collaborative robotics

**DOI:** 10.3389/frobt.2025.1623884

**Published:** 2025-12-04

**Authors:** Davide De Lazzari, Matteo Terreran, Giulio Giacomuzzo, Siddarth Jain, Pietro Falco, Ruggero Carli, Stefano Ghidoni, Diego Romeres

**Affiliations:** 1 Department of Information Engineering, University of Padua, Padua, Italy; 2 Mitsubishi Electric Research Laboratories, Cambridge, MA, United States

**Keywords:** open-end dynamic time warping, human action progress estimation, human action completion time prediction, human-robot interaction, collaborative assembly, real-time monitoring, reinforcement learning, sliding window cross-correlation

## Abstract

Real-time estimation of human action progress is critical for seamless human-robot collaboration yet remains underexplored. With this paper we propose the first real-time application of Open-end Soft-DTW (OS-DTW_EU_) and introduce OS-DTW_WP_, a novel DTW variant that integrates a Windowed-Pearson distance to effectively capture local correlations. This method is embedded in our Proactive Assistance through action-Completion Estimation (PACE) framework, which leverages reinforcement learning to synchronize robotic assistance with human actions by estimating action completion percentages. Experiments on a chair assembly task demonstrate OS-DTW_WP_’s superiority in capturing local motion patterns and OS-DTW_EU_’s efficacy in tasks presenting consistent absolute positions. Moreover we validate the PACE framework through user studies involving 12 participants, showing significant improvements in interaction fluency, reduced waiting times, and positive user feedback compared to traditional methods.

## 1 Introduction

In dynamic Human-Robot Collaboration (HRC), the ability to perceive and predict human actions in real time is foundational to achieving seamless coordination. Whether ensuring safety in shared workspaces, minimizing idle times in assembly tasks, or adapting to operator preferences, robots must continuously monitor human progress to act as responsive partners rather than rigid tools. Existing approaches often rely on predefined task sequences or assume idealized human behavior, limiting their applicability in real-world scenarios where operators exhibit variability in motion speed, style, and decision-making. Without robust progress estimation, robots risk desynchronization—delaying assistance, causing interruptions, or even compromising safety.

This paper addresses a core challenge in HRC: real-time estimation of human action progress and prediction of action completion time; enabling robots to synchronize their motions with human workflows at the level of individual actions. While existing research often focuses on high-level task planning or post-hoc activity recognition, the ability to track the progression in real time of atomic human actions (e.g., picking up a screwdriver, inserting a component), which is critical for coordination, remains underexplored. Consider collaborative assembly: if a robot misjudges the completion of a human operator’s action, such as tightening a screw, it may prematurely retrieve the next part (disrupting focus) or delay assistance (introducing idle time). These errors, though seemingly minor, compound across workflows, eroding efficiency and trust.

### 1.1 Contributions

To address the previous challenges, our work advances the state of the art across three interrelated dimensions. First, we introduce novel online Dynamic Time Warping (DTW) variants for real-time human progress estimation. Second, we demonstrate that these techniques enable the precise prediction of remaining action durations. Finally, we integrate these methods into a collaborative assembly framework designed to minimize idle times and ensure seamless synchronization between robot and human operator.

Our methodological contributions include:

- We introduce OS-DTW_WP_, a novel open-ended DTW approach for real-time human progress estimation that incorporates a Windowed-Pearson (WP) distance. We formalize the WP distance as a shape descriptor within the shapeDTW framework (Zhao and Itti, 2018), analyze its computational complexity, and present an optimized implementation for real-time operation.
- We propose two distinct methods for predicting action completion times, along with a hybrid approach that synergistically combines their strengths while mitigating individual limitations.
- We present the Proactive Assistance through action-Completion Estimation (PACE) framework—a Reinforcement Learning-based system that leverages continuous human progress monitoring to synchronize proactive robot assistance with human operators, explicitly reducing waiting times through predictive scheduling.

We validate our approach through real-world experiments involving a chair assembly task with human participants, by tracking their hand motions. Yielding the following experimental contributions:

- We provide empirical evidence that classical Open-end DTW is inadequate for handling human motion variability, whereas our Open-end Soft-DTW implementation—which we denote as OS-DTW_EU_ given its reliance on the Euclidean distance—demonstrates robust performance. To our knowledge, this represents the first real-time application of Open-end Soft-DTW.
- We quantitatively show that OS-DTW_WP_ overcomes the failure cases observed with OS-DTW_EU_ while maintaining relatively strong performance across diverse motion patterns. Our analysis further indicates that similar limitations are present in (offline) Soft-DTW, which can be effectively mitigated by incorporating the Windowed-Pearson distance.
- We demonstrate the effectiveness of our completion-time estimation methods, which outperform previous approaches based on Open-end DTW.
- We validate the PACE framework through real-user experiments, highlighting the efficacy of OS-DTW_WP_ in improving collaborative efficiency as evidenced by both quantitative metrics and subjective evaluations.

This work builds on a prior conference publication (De Lazzari et al., 2025). This work significantly extends our prior publication through key methodological and experimental enhancements. Methodologically, we formally establish the Windowed-Pearson (WP) distance as a shape descriptor within the shapeDTW framework, bridging theoretical foundations with practical applications. We further analyze OS-DTW_WP_’s computational complexity and present its optimized implementation for real-time deployment, critical considerations omitted previously. The temporal forecasting methodology, encompassing nominal, linear, and hybrid approaches, is entirely novel, as our prior work focused solely on progress estimation without duration prediction. Additionally, we detail the initialization procedure for the simulated environment used to train the PACE policy. Experimentally, we present new analyses comparing online DTW variants to expose their limitations, along with comprehensive evaluations of completion time estimation methods. We also include PACE training results and simulate additional methods using newly collected collaborative assembly demonstrations. Beyond these extensions, this work provides in-depth technical discussions, including refined literature comparisons, theoretical justifications for design choices, expanded failure case analyses, and detailed evaluations of time estimation effectiveness across diverse experimental conditions.

### 1.2 Related works

#### 1.2.1 HRC frameworks

Human-robot collaboration (HRC) demands systems capable of dynamically adapting to human actions while maintaining safety and efficiency. Early approaches focused on optimizing task sequencing (Chen et al., 2013; Rahman et al., 2015) by pre-assigning roles to humans or robots, resulting in rigid workflows. While effective in controlled settings, such methods struggle to accommodate real-world variability in human motion and decision-making. Subsequent work adopted leader-follower paradigms, where robots reactively adjust actions based on predefined human workflows (Cheng et al., 2020; Cheng et al., 2021; Ramachandruni et al., 2023; Giacomuzzo et al., 2024). However, empirical studies reveal that human operators prefer retaining task control while also expecting robots to anticipate their needs proactively (Lasota and Shah, 2015). This necessitates real-time monitoring of human actions to enable predictive assistance—a capability absent in existing task-allocation frameworks. Critically, none of these methods actively monitor human actions during execution, limiting their ability to recognize and synchronize with ongoing activities.

#### 1.2.2 Real-time human progress estimation

Beyond HRC, a rich body of research has investigated human motion analysis, particularly focusing on action recognition (Mao et al., 2023; Yan et al., 2018; Ray et al., 2025) and motion prediction (Mao et al., 2020; Dang et al., 2021; Chen et al., 2023). These methods rely on estimated human joint positions, obtained from vision or inertial sensors, to classify actions or predict motion trajectories. While highly effective on activity recognition benchmarks and gesture-level prediction tasks, the majority of these approaches are designed for offline analysis rather than real-time deployment. A few exceptions demonstrate online operation (Chi et al., 2025; An et al., 2023), but even these focus primarily on recognizing discrete actions in streaming settings. Consequently, they do not provide continuous estimates of human task progression during execution, which is essential for action completion estimation in collaborative scenarios.

Real-time human progress monitoring remains an underexplored topic. To our knowledge, only two approaches that estimate the human progress at the action level have been proposed: Maderna et al. (2019), Maderna et al. (2020) employ Open-end Dynamic Time Warping (OE-DTW) (Sakoe, 1979) to estimate human progression, while Cheng and Tomizuka (2021) propose a Sigma log-normal model for predicting action completion times. The latter reports superior performance over DTW in their evaluations, however, our analysis reveals that OE-DTW, while providing temporal flexibility through nonlinear alignment, suffers from oversensitivity to trajectory shape variations common in real-world human motions. A follow-up work by the same authors (Leu et al., 2022) applies the same method for task planning in a collaborative assembly application.

#### 1.2.3 Dynamic time warping

Dynamic Time Warping (DTW) (Sakoe and Chiba, 1978) is a well-known method for computing similarity between temporally misaligned sequences. Open-end DTW (Sakoe, 1979) relaxes endpoint constraints, enabling partial matches for causal systems. While Maderna et al. (2019) applied OE-DTW for real-time progress estimation, our experiments show its unsuitability to the variability of human actions with extensive real-user studies. We address this limitation through an open-ended variant of Soft-DTW (Cuturi and Blondel, 2017), which replaces DTW’s hard min operator with a differentiable softmin to mitigate local minima. Though open-ended Soft-DTW has shown promise for offline skeleton-based recognition (Manousaki and Argyros, 2023), its application in real-time human progress monitoring remains unexplored.

A deeper limitation persists: standard DTW variants use Euclidean distance, which prioritizes absolute spatial alignment over shape similarity. This proves problematic when human motions preserve geometric structure but vary in speed or amplitude.

Recent works focus on on task-adaptive time warping (Trigeorgis et al., 2016; Matsuo et al., 2023), particularly for aligning machine learning datasets. Trigeorgis et al. (2016) learns complex non-linear representations of multiple time-series based on canonical correlation analysis, while Matsuo et al. (2023) learns a distance metric by training an attention model. However, these methods require large training datasets and full knowledge of the signals, making them incompatible with open-ended scenarios where future data are unknown.

Correlation Optimized Warping (COW) (Nielsen et al., 1998) offers an alternative by maximizing Pearson correlation between signal segments, to match similar segments in fields like chromatography, proteomics, and seismology. However, COW’s rigid windowing sacrifices DTW’s temporal elasticity (Tomasi et al., 2004). Recently, seismological research (Wang et al., 2023) has combined DTW with a windowed correlation-based distance, implementing an offline, one-dimensional approach using a weighted biased cross-correlation distance with windows centered on each sample. While this marks an initial attempt to integrate correlation analysis with DTW, their method fundamentally differs from our requirements for real-time human monitoring.

Our method addresses these gaps by adapting Soft-DTW for real-time open-ended alignment (OS-DTW_EU_), overcoming the practical limitations of OE-DTW. Additionally, we introduce the Windowed-Pearson (WP) distance, which computes local Pearson correlations within sliding windows along the trajectory. Unlike COW’s segment-wise approach, WP integrates shape similarity into the DTW framework, enabling both local and global optimal alignment. This combination (OS-DTW_WP_) ensures invariance to absolute position shifts and effectively captures local patterns while preserving temporal flexibility–an essential feature for HRC applications, where humans may perform similar motions with varying locations, speeds, and intensities.

### 1.3 Paper outline

The remainder of the paper is organized as follows. In Section 2, we describe our proposed methods. We begin by introducing preliminaries on existing Dynamic Time Warping algorithms in Section 2.1, with a focus on the Open-end Soft-DTW algorithm. Next, in Section 2.2, we present the OS-DTW_WP_ algorithm for real-time phase estimation, including its implementation and parameter tuning. In Section 2.3, we propose and analyze three distinct methods for action completion time prediction using online DTW. Following this, in Section 2.4, we describe the PACE framework, formulating the problem as a Partially Observable Markov Decision Process and detailing the derivation of a simulated environment for policy training. In Section 3, we outline the experimental procedure. In Section 4, we present the results on phase estimation, action completion time estimation, and collaborative assembly. In Section 5 we discuss our findings and suggest directions for future work. Finally, Supplementary Material provides further details on the Dynamic Time Warping algorithms employed in the experiments.

## 2 Methods

### 2.1 Preliminaries

#### 2.1.1 Notation

For a vector, sequence, or signal a , we denote by $a_{i}$ the element at index i (with indices starting from 0). For a matrix A , $A_{i,j}$ refers to the element in row i and column j with 0-based indexing. Given a vector a , the subvector from index i to j (inclusive) is denoted by $a_{i:j}$ . Submatrices are defined analogously.

#### 2.1.2 Dynamic time warping

Dynamic Time Warping (DTW) (Sakoe and Chiba, 1978) is an algorithm designed to compute the optimal alignment between two time-dependent sequences that may vary in speed, enabling a flexible, nonlinear temporal mapping. Typically, DTW enforces that the warping path starts at the first index and ends at the last index of both sequences. Moreover, each index in one sequence is matched with one or more indices in the other, subject to continuity and monotonicity constraints that preserve the original temporal order. The algorithm outputs both the *temporal alignment* (commonly referred to as the *warping path*), and the *alignment cost*, also known as the *DTW cost*.

DTW is inherently an asymmetric algorithm, designating one sequence as the *reference*, $b=\left[b_{0},\ldots ,b_{n-1}\right]\in R^{n\times d}$ , and the other as the *query* or *test* sequence, $a=\left[a_{0},\ldots ,a_{m-1}\right]\in R^{m\times d}$ . In this paper, we use the terms *sequences*, *signals*, and *trajectories* interchangeably. Additionally, the DTW algorithms we treat in this paper are generalized to handle multidimensional signals and designed to output the *phase* $\tau =\left[\tau_{0},\ldots ,\tau_{m-1}\right]$ of the query trajectory with respect to the reference trajectory. The *phase* of a signal, is defined as the normalized progress along a reference trajectory. Each point $a_{i}$ in the query sequence is matched with a point $b_{j_{i}}$ in the reference. The phase $\tau_{i}$ of a at i is then defined as:

$$\tau_{i}=\frac{j_{i}}{n-1}\in \left[0,1\right].$$

Moreover, while classical DTW uses a pointwise Euclidean (or squared Euclidean) distance, the reported algorithms generalize to an arbitrary distance function δ(⋅,⋅) .

Dynamic Time Warping involves three key steps:

- Distance matrix computation: A matrix D is computed to have the distances between all pairs of points of the reference and query sequences.
- Forward recursion: Dynamic programming is used to compute the minimum cumulative cost to reach each point in the matrix through a path. This is obtained by computing a matrix of the cumulative cost R .
- Backward recursion: The optimal warping path is traced from the last point to the start.

**FIGURE 1:**
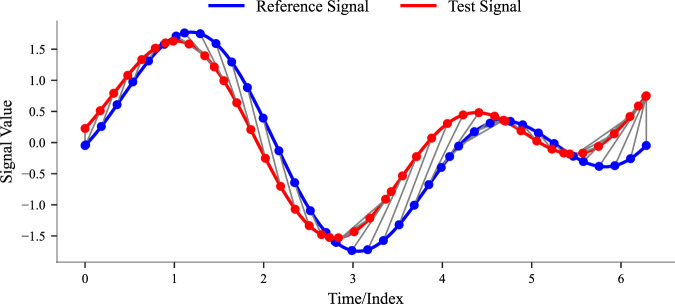
Illustrative example of an alignment between two similar signals obtained with Dynamic Time Warping.

The detailed algorithm is reported in the Supplementary Material (see Supplementary Algorithm 1), while a visual representation of the DTW alignment is shown in Figure 1.

#### 2.1.3 Open-end DTW

Classical DTW requires the reference and entire query sequence to compute the optimal alignment. This requirement makes DTW unsuitable for real-time applications when sequences are streamed, as it would need access to future data points to compute the alignment.

Open-end DTW, first employed by Sakoe (1979), is a variant that relaxes the constraint that the last point of the two sequences should match, and computes the alignment which best matches all of the query with a first section of the reference. To do so, after the computation of the cumulative cost matrix R , the index of the reference sequence point, $b_{j^{*}}$ , matching with the last point of the query, is chosen as the one with the minimal cost, namely,

$$j^{*}=\underset{j\in \left[0,n-1\right]}{\mathrm{arg min}}R_{m-1,j}.$$

Classical DTW, which we will refer to as *offline* DTW to distinguish it from Open-end DTW, matches the entire sequences, enabling the direct derivation of the phase from the full alignment, with the ability to reference both past and future data from the sequences. In contrast, Open-end DTW can operate on a partially available query trajectory. Although a recursive process could be employed to estimate the alignment of the query trajectory to the truncated reference, this paper focuses on real-time phase estimation, which makes the recursive step unnecessary. Specifically, the phase at time step i∈[0,m−1] is estimated causally using only past and current query data, without requiring future information. The phase estimate at step i is given by:

$$\tau_{i}=\frac{j^{*}}{n-1}.$$

#### 2.1.4 Open-end soft-DTW

DTW is effective at aligning signals that vary in speed; however, by penalizing both minor and major time shifts equally, it can sometimes produce unrealistic warping paths. This drawback is even more pronounced in Open-end DTW, where no constraint exists to ensure that the final samples of the two signals match.

For offline DTW, this problem has been addressed using path constraints, such as the Sakoe-Chiba Band (Sakoe and Chiba, 1978) and the Itakura Parallelogram (Itakura, 1975), or weighting schemes like Weighted DTW (Jeong et al., 2011), which penalize warping paths deviating from the diagonal. However, these methods are not directly applicable in an online scenario, as the diagonal is unknown.

Soft-DTW (Cuturi and Blondel, 2017) replaces the minimum operation in the forward recursion of the DTW algorithm with a soft-minimum, making the DTW loss differentiable. Specifically, the min operator in the forward recursion of DTW, see Supplementary Algorithm 1 Step 5, is replaced by:

$$\min^{\gamma }\left(a,b,c\right)=\left\{\begin{array}{cc} -\gamma \log\left(e^{-a/\gamma }+e^{-b/\gamma }+e^{-c/\gamma }\right),\; & \;\gamma >0,\\ \min\left(a,b,c\right),\; & \;\gamma =0. \end{array}\right.$$

to ensure numerical stability when γ>0 , the soft-minimum is calculated using the *log-sum-exp trick*:

$$\min^{\gamma }\left(a,b,c\right)=-\gamma \left(\log\left(e^{-\left(a-\mu \right)/\gamma }+e^{-\left(b-\mu \right)/\gamma }+e^{-\left(c-\mu \right)/\gamma }\right)+\frac{\mu }{\gamma }\right),\;\text{ if }\gamma >0$$

where μ=max(a,b,c) .

Originally designed for time series averaging and clustering, Soft-DTW introduces a smoothing factor that helps mitigate local minima. In particular, the soft-minimum weighs all possible paths, ensuring that slight distortions do not dominate the final alignment. With γ=0 , the formulation reduces to the standard minimum operation, whereas γ→∞ results in a cumulative cost equal to the sum of all costs. This formulation allows Soft-DTW to handle temporal variability more effectively. In fact, Janati et al. (2020) show that the Soft-DTW loss is not invariant to time shifts and grows quadratically with respect to the time shift, making it suitable for open-end signal matching.

For completeness, we report a version of the Open-end Soft-DTW algorithm for causal phase estimation in Algorithm 1.

Algorithm 1 Open-end Soft-DTW.

 **Inputs:**
  - Query signal $a=\left[a_{0},\ldots ,a_{m-1}\right]\in R^{m\times d}$

  - Reference signal $b=\left[b_{0},\ldots ,b_{n-1}\right]\in R^{n\times d}$

  - Distance δ(⋅,⋅)

  - Smoothing parameter γ≥0

 **Output:**
  - Estimated phase $\tau =\left[\tau_{0},\ldots ,\tau_{m-1}\right]\in R^{m}$ of a w.r.t. b

 1: Initialize $D\in R^{m\times n}$ , where $D_{i,j}=\delta \left(a_{i},b_{j}\right)$ ⊳ Distance matrix computation
 2: Initialize $R\in R^{\left(m+1\right)\times \left(n+1\right)}$ , with $R_{0,0}=0$ , $R_{i,0}=\infty$ for i∈[1,m] , ⊳ Forward recursion and $R_{0,j}=\infty$ for j∈[1,n]

 3: **for** i=1 to m **do**
 4:  **for** j=1 to n **do**
 5: $R_{i,j}=D_{i-1,j-1}+\min^{\gamma }\left(R_{i-1,j},R_{i,j-1},R_{i-1,j-1}\right)$

 6:  **end for**
 7: $j*={\mathrm{arg min}}_{j\in \left[0,n-1\right]}R_{i,j}$

 8: $\tau_{i}=j*/\left(n-1\right)$

 9: **end for**

### 2.2 Online DTW for real-time phase estimation

#### 2.2.1 Windowed-Pearson distance as a DTW metric

While the Euclidean distance remains the default metric to measure sample-wise similarity in Dynamic Time Warping, this metric assumes consistent absolute scaling between signals. Though Opend-end Soft-DTW is effective for signals with consistent absolute magnitudes and baseline positions, its reliance on the Euclidean distance makes it sensitive to vertical offsets and amplitude variations, often producing suboptimal warping paths for signals that share geometric structure but differ in execution scale.

Recent approaches like shapeDTW (Zhao and Itti, 2018) address this limitation by converting raw signals into shape descriptors (e.g., piecewise aggregate approximations or discrete wavelet coefficients) prior to alignment.

Inspired by correlation analysis, our approach adapts this shape-sensitive philosophy by introducing a windowed Pearson distance that locally normalizes amplitude differences during alignment. This creates an online-capable method that directly compares trajectory shapes through local correlation analysis, while maintaining DTW’s temporal elasticity. The combination of windowed normalization with open-end alignment proves particularly effective for human-robot collaboration scenarios, where human motion patterns exhibit consistent geometric features but significant trial-to-trial variability in speed and scale.

We formally define the Windowed-Pearson (WP) distance between two signal samples as:

$$\delta_{\text{WP}}^{w}\left(a_{i},b_{j}\right)≔\overset{d-1}{\underset{k=0}{\sum }}\left(1-\frac{\mathrm{Cov}\left(a_{i-w+1:i,k},\;b_{j-w+1:j,k}\right)}{\sqrt{\mathrm{Var}\left(a_{i-w+1:i,k}\right)\mathrm{Var}\left(b_{j-w+1:j,k}\right)}}\right).$$ (1)

to calculate the distance when i<w+1 or j<w+1 , we pad the signals with their initial values.

Note that in the one-dimensional case, this distance reduces to the Pearson distance between two segments $a_{i-w+1:i}$ and $b_{j-w+1:j}$ . Thus it can be rewritten as s

$$\delta_{\text{WP}}^{w}\left(a_{i},b_{j}\right)=\overset{d-1}{\underset{k=0}{\sum }}\left(1-\rho \left(a_{i-w+1:i,k},b_{j-w+1:j,k}\right)\right)$$

where ρ(⋅,⋅) denotes the Pearson correlation coefficient between two segments.

By design, the WP distance is invariant to vertical shifts and can effectively capture local correlations. A small window measures similarity between samples based on fine-grained local patterns, while a larger window captures broader, more extended patterns.

Furthermore, we demonstrate that for one-dimensional signals, this distance is equivalent to employing *z-normalization* as the mapping function to calculate the shape descriptor on a window w , as defined by Zhao and Itti (2018).

Consider two windowed segments a and b of length w . Applying *z-normalization*, we obtain:

$$\tilde{a}=\frac{a-\bar{a}}{\sigma_{a}},\;\tilde{b}=\frac{b-\bar{b}}{\sigma_{b}},$$

where $\bar{a}$ , $\bar{b}$ , and $\sigma_{a}$ , $\sigma_{b}$ , denote the means and standard deviations of segments a and b , respectively. For z-normalized signals, it holds that:

$$\overset{w-1}{\underset{i=0}{\sum }}{\tilde{a}}_{i}^{2}=\overset{w-1}{\underset{i=0}{\sum }}{\tilde{b}}_{i}^{2}=w,$$

and

$$\rho \left(a,b\right)=\frac{1}{w}\overset{w}{\underset{i=1}{\sum }}{\tilde{a}}_{i}{\tilde{b}}_{i}.$$ (10)

therefore the squared Euclidean distance between the two normalized segments becomes:

$$\|\tilde{a}-\tilde{b}{\|}_{2}^{2}=\overset{w}{\underset{i=1}{\sum }}\left({\tilde{a}}_{i}^{2}+{\tilde{b}}_{i}^{2}-2{\tilde{a}}_{i}{\tilde{b}}_{i}\right)=2w\left(1-\rho \left(a,b\right)\right),$$

where the final equality follows from substituting the two previous equalities.

Thus, for z-normalized segments, minimizing the squared Euclidean distance is equivalent to minimizing the Pearson distance (up to the multiplicative constant 2w ). This makes the WP distance defined in Equation 1 a suitable mapping function for shapeDTW.

#### 2.2.2 Parameter tuning

OS-DTW_EU_ and OS-DTW_WP_ require the tuning of one and two parameters, respectively. Specifically, the smoothing factor γ≥0 and, for OS-DTW_WP_, also the window size w∈[1,2,3,… ] .

Assuming access to a training dataset, these parameters can be optimized to minimize the average mean squared error (MSE) between the estimated phase and a ground truth phase. An effective choice for the ground truth is a linear phase evolution, defined as:

$${\bar{\tau }}_{i}=\frac{i}{m-1}\;\text{for}\;i\in \left[0,m-1\right].$$

Alternatively, the ground truth phase can be computed using offline DTW methods such as Soft-DTW.

In scenarios where the ultimate goal is to minimize a cost function that depends on the phase, the cost function itself can serve as the optimization objective.

While various optimization methods are applicable, we select Bayesian Optimization (Snoek et al., 2012) as our preferred method, as it requires a small number of evaluations of the cost function.

#### 2.2.3 Real-time implementation

Open-end Soft-DTW, as described in Algorithm 1, is not directly suitable for real-time applications. To address this limitation, modifications are necessary to handle streaming input signals efficiently and to store information in a manner that ensures constant computational complexity at each step, thereby enabling bounded-time computation. Such an adapted algorithm is presented in Algorithm 2.

When a new sample $a_{i}$ arrives, the algorithm first computes d , the i -th row of the distance matrix D (as defined in Algorithm 1), which represents the distances between the new sample and all reference samples. Subsequently, r is computed, corresponding to the i -th row of the accumulated cost matrix R (as defined in Algorithm 1) to update the warping costs.

Algorithm 2 Online Open-end Soft DTW.

 **Inputs:**
  - Streaming signal $a_{i}\in R^{d}$ sampled at each time step i∈[0,1,… ] from the query trajectory $a=\left[a_{0},a_{1},\ldots \;\right]$

  - Reference trajectory $b=\left[b_{0},\ldots ,b_{n-1}\right]\in R^{n\times d}$

  - Distance δ(⋅,⋅)

  - Smoothing parameter γ≥0

 **Output:**
  - Continuous output ${\hat{\tau }}_{i}$ at each time step i=0,1,… 

 1: Initialize $d\in R^{n}$

 2: Initialize $r\in R^{n+1}$ , with $r_{0}=0$ , $r_{j}=\infty$ for j∈[1,n]

 3: Initialize $r^{\prime }\in R^{n+1}$ , with $r_{0}=\infty$

 4: **while** there is a new sample $a_{i}$ **do**
 5:  **for** j=0 to n−1 **do** ⊳ Distance computation
 6: $d_{j}=\delta \left(a_{i},b_{j}\right)$

 7:  **end for**
 8:  **for** j=1 to n **do** ⊳ One-step forward recursion
 9: $r_{j}^{\prime }=d_{j-1}+\min^{\gamma }\left(r_{j},r_{j-1}^{\prime },r_{j-1}\right)$

 10:  **end for**
 11:  Set $r=r^{\prime }$

 12:  Output ${\hat{\tau }}_{i}={\mathrm{arg min}}_{j\in \left[0,n-1\right]}r_{j}/\left(n-1\right)$

 13: **end while**

This real-time version only requires storing the last computed rows of matrices D and R , resulting in a constant O(n) space and time complexity. This represents a significant improvement over the O(m⋅n) complexity of the “offline” version described in Algorithm 1. The overall complexity depends also on the computational cost of the distance function δ , which is O(d) for the Euclidean distance (where d denotes the number of dimensions of the signals) and O(d⋅w) for the WP distance (where w represents the window size). Consequently, the per-step time complexity is O(n⋅d) for OS-DTW_EU_ and O(n⋅d⋅w) for OS-DTW_WP_.

Although each step has constant computational complexity, an optimized implementation is crucial not only for real-time applications but also for offline scenarios where many long sequences need be processed. For brevity, we describe in detail the optimized implementation of Algorithm 1 and then briefly explain how it can be adapted to the real-time version (Algorithm 2).

First, we note that computing the distance matrix requires m⋅n evaluations of δ . These operations are mutually independent and thus inherently parallelizable. However, this parallelism does not extend to the subsequent recursion steps, where code efficiency becomes critical. In particular, pre-compilation of this stage can yield significant computational benefits. Given the simplicity of the recursive operations, such optimization is sufficient to ensure real-time feasibility.

When δ is the Euclidean distance, each evaluation has O(d) complexity, where d denotes the number of dimensions. The entire distance matrix D can be computed efficiently through vectorized operations across the n and m dimensions. For the WP distance, each evaluation has O(d⋅w) complexity (where w represents the window size), however, standard scientific computing libraries like numpy and scipy lack built-in support for vectorized computation of this metric.

Calculating the entire D matrix requires computing the correlation between all possible pairs of windows. To achieve this, we construct matrices ${\tilde{A}}_{k}\in R^{w\times m}$ and ${\tilde{B}}_{k}\in R^{w\times n}$ for each dimension k∈[0,d−1] , containing all possible windows of the respective signals. Specifically:

$${\tilde{A}}_{k}=\left[\begin{array}{cccccc} a_{0,k} & a_{1,k} & a_{2,k} & a_{3,k} & \cdots & a_{m-1,k}\\ a_{0,k} & a_{0,k} & a_{1,k} & a_{2,k} & \cdots & a_{m-2,k}\\ a_{0,k} & a_{0,k} & a_{0,k} & a_{1,k} & \cdots & a_{m-3,k}\\ \vdots & \vdots & \vdots & \ddots & \ddots & \vdots \\ a_{0,k} & a_{0,k} & a_{0,k} & \cdots & a_{m-w+2,k} & a_{m-w+1,k}\\ a_{0,k} & a_{0,k} & a_{0,k} & \cdots & a_{m-w+1,k} & a_{m-w,k} \end{array}\right],$$

and similarly for ${\tilde{B}}_{k}$ .

Next, we compute the column-wise covariance $C^{k}=\text{Cov}\left({\tilde{A}}_{k},{\tilde{B}}_{k}\right)$ using an efficient method (e.g. numpy.cov). The resulting matrix $C^{k}\in R^{\left(m+n\right)\times \left(m+n\right)}$ satisfies:

$$C_{i,j}^{k}=\text{Cov}\left(a_{i-w:i,k},b_{j-w:j,k}\right).$$

The top-left submatrix (of size m×m ) represents the covariance between columns of ${\tilde{A}}^{k}$ . The bottom-right submatrix (of size n×n ) represents the covariance between columns of ${\tilde{B}}^{k}$ . The top-right and bottom-left submatrices represent the covariance between columns of ${\tilde{A}}^{k}$ and columns of ${\tilde{B}}^{k}$ .

The matrix D can then be computed efficiently by performing standard operations on these submatrices.

For the online version reported in Algorithm 2, ${\tilde{B}}_{k}$ can be computed offline, and for each new sample $a_{i}$ we compute the column-wise covariance between ${\tilde{B}}_{k}$ and the vector $a_{i-w:i,k}$ .

Moreover, to ensure numerical stability we add a small value $\left(10^{-12}\right)$ to the variances at the denominator of the WP distance in Equation 1.

### 2.3 Action completion time prediction with online DTW

#### 2.3.1 Nominal and linear estimation methods

We present two approaches for predicting the completion time of a human action based on the real-time phase estimate provided by OS-DTW. This phase estimate offers valuable insight into the current progress of an action, allowing us to forecast its eventual completion.

The first approach assumes that the user will maintain their current pace, using the observed execution speed to estimate the remaining time. The second approach relies on a nominal execution pace derived from historical demonstrations. Both methods, calibrated with prior data, can enable systems to estimate the future duration of a human action—a capability critical for applications such as assistive robotics and collaborative tasks that require timely intervention.

We assume access to a reference trajectory and a set of training trajectories, each of which is d -dimensional and sampled at a constant time step $T_{s}$ . For convenience, we define $t_{\text{end}}\left(y\right)$ as the function returning the completion time of a trajectory y , and $\phi_{y}\left(t\right)$ as the function returning the estimated phase τ for the same trajectory at time t .

The *nominal duration* $\bar{t}$ is defined as the average duration of all training trajectories and the reference trajectory:

$$\bar{t}=E_{y}\left[t_{\text{end}}\left(y\right)\right].$$

Thus, the best a-priori estimate for the completion time, based solely on prior information and the current time, is:

$${\hat{t}}_{o}\left(t\right)=\max\left(\bar{t},t\right).$$

We define the *nominal* estimation method as

$${\hat{t}}_{\text{nom}}\left(t,\tau \right)=t+\left(1-\tau \right)\;\bar{t},$$

and the *linear* estimation method as

$${\hat{t}}_{\text{lin}}\left(t,\tau \right)=\frac{t}{\tau },$$

where $t\in R^{\geq 0}$ is the current time and τ∈[0,1] is the estimated phase.

The *nominal* method assumes execution progresses at the nominal speed, with the remaining time estimated as $\left(1-\tau \right)\;\bar{t}$ . The *linear* method assumes a constant execution speed, scaling the current time t inversely with the estimated completion percentage τ . The *linear* method is analogous to that employed by Maderna et al. (2019).

#### 2.3.2 Hybrid estimation method

The linear estimation method is highly sensitive to phase miscalculations and can be inaccurate when the available trajectory percentage is insufficient to reliably estimate future execution speed. Conversely, the nominal estimation method does not leverage past execution speed as an informative metric for predicting the completion time. To address these limitations, we derive an optimal switching rule based on the estimated phase and elapsed time to transition from the nominal to the linear estimation method.

We begin by calculating the optimal switching time. The mean absolute estimation error (MAE) at time t for the nominal estimation method is defined as

$${\text{MAE}}_{\text{nom}}\left(t\right)=E_{y}\left[\left|{\hat{t}}_{\text{nom}}\left(t,\phi_{y}\left(t\right)\right)-t_{\text{end}}\left(y\right)\right|\right],$$

and for the linear estimation method as

$${\text{MAE}}_{\text{lin}}\left(t\right)=E_{y}\left[\left|{\hat{t}}_{\text{lin}}\left(t,\phi_{y}\left(t\right)\right)-t_{\text{end}}\left(y\right)\right|\right].$$

To determine the optimal switching time, we calculate the cumulative nominal costs up to each time t and the cumulative linear costs from each time t up to the maximum final time $t_{\text{max}}=\underset{y}{\max}t_{\text{end}}\left(y\right)$ :

$$C_{\text{nom}}\left(t\right)=\underset{k\in \left[0,t\right]}{\sum }{\text{MAE}}_{\text{nom}}\left(k\right),$$

$$C_{\text{lin}}\left(t\right)=\underset{k\in \left[t+T_{s},t_{\text{max}}\right]}{\sum }{\text{MAE}}_{\text{lin}}\left(k\right).$$

The total cost for switching at time t is given by:

$$C_{\text{total}}\left(t\right)=C_{\text{nom}}\left(t\right)+C_{\text{lin}}\left(t\right).$$

Thus, the optimal switching time t* is:

$$t^{*}=\underset{t\in \left[0,t_{\text{max}}\right]}{\mathrm{arg min}}\;C_{\text{total}}\left(t\right).$$

A similar procedure can be applied to estimate the optimal switching phase. We define the MAE for a phase τ using the nominal and linear estimation methods respectively as:

$${\text{MAE}}_{\text{nom}}^{\prime }\left(\tau \right)=E_{y}\left[\left|{\hat{t}}_{\text{nom}}\left(\phi_{y}^{-1}\left(\tau \right),\tau \right)-t_{\text{end}}\left(y\right)\right|\right],$$

$${\text{MAE}}_{\text{lin}}^{\prime }\left(\tau \right)=E_{y}\left[\left|{\hat{t}}_{\text{lin}}\left(\phi_{y}^{-1}\left(\tau \right),\tau \right)-t_{\text{end}}\left(y\right)\right|\right].$$

The cumulative costs are then:

$$C_{\text{nom}}^{\prime }\left(\tau \right)=\int_{0}^{\tau }{\text{MAE}}_{\text{nom}}^{\prime }\left(z\right)\;dz,$$

$$C_{\text{lin}}^{\prime }\left(\tau \right)=\int_{\tau }^{1}{\text{MAE}}_{\text{lin}}^{\prime }\left(z\right)\;dz.$$

The total cost for switching at phase τ is given by:

$$C_{\text{total}}^{\prime }\left(\tau \right)=C_{\text{nom}}^{\prime }\left(\tau \right)+C_{\text{lin}}^{\prime }\left(\tau \right).$$

The optimal switching phase τ* is:

$$\tau^{*}=\underset{\tau \in \left[0,1\right]}{\mathrm{arg min}}\;C_{\text{total}}^{\prime }\left(\tau \right).$$

### 2.4 Online DTW for proactive assistance in human-robot collaboration

**FIGURE 2:**
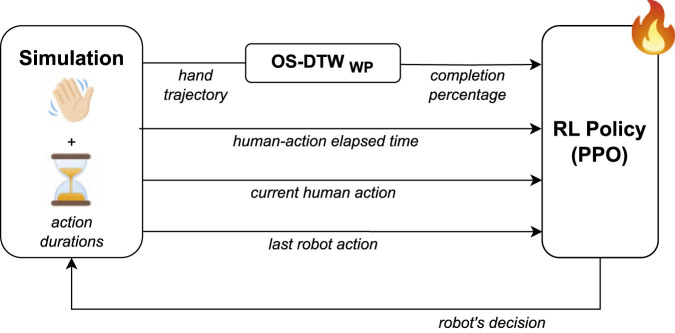
PACE training scheme. Demonstrations of the collaborative assembly task are first recorded, including human hand trajectories and action durations. These data are used to set up a simulation (left), which models the task as a POMDP. A policy (right) is then trained with proximal policy optimization, using as inputs: (i) the human action completion percentage (estimated by OS-DTW_WP_), (ii) the elapsed time of the current human action, (iii) the current human action, and (iv) the last robot action.

In this subsection, we propose the application of OS-DTW_WP_ in a human-robot collaboration setting. We introduce the *Proactive Assistance through action-Completion Estimation* (PACE) framework, which leverages the estimated phase of human actions to synchronize the robot’s behavior with the human’s workflow in a collaborative assembly task. The goal of PACE is to minimize idle times for both the human and the robot, ensuring efficient and seamless collaboration. A depiction of the PACE training scheme is provided in Figure 2.

PACE models the system as a Partially Observable Markov Decision Process (POMDP) (Kaelbling et al., 1998), and utilizes data collected from human demonstrations and OS-DTW_WP_ to create a simulated environment and train a policy via Reinforcement Learning (RL). This approach enables direct training with the estimated phase, eliminating the need to explicitly compute action completion times as an intermediate step.

#### 2.4.1 Collaborative problem formulation

We consider a scenario where a human and a robotic manipulator concurrently perform separate tasks. The robot executes a sequence of M *robot-task* actions $R={\left\{a_{i}^{R}\right\}}_{i=1}^{M}$ , which are repeated indefinitely. Simultaneously, the human undertakes a sequence of N *human* actions, denoted as $H={\left\{a_{j}^{H}\right\}}_{j=1}^{N}$ . The human requires the robot’s assistance to complete a subset of these actions, referred to as *joint* actions and represented by $J={\left\{a_{l}^{J}\right\}}_{l=1}^{L}$ , where J⊆H .

To formalize the relationship between joint and human actions, we define the operator α(⋅) to map the index of a joint action to the corresponding human action index, such that $a_{\alpha \left(l\right)}^{H}=a_{l}^{J}$ . For simplicity, we assume the last human action is a joint action (i.e., $a_{N}^{H}=a_{L}^{J}$ ), and no two consecutive human actions are joint actions (i.e., if $a_{j}^{H}\in J$ , then $a_{j+1}^{H}\notin J$ ).

**FIGURE 3:**
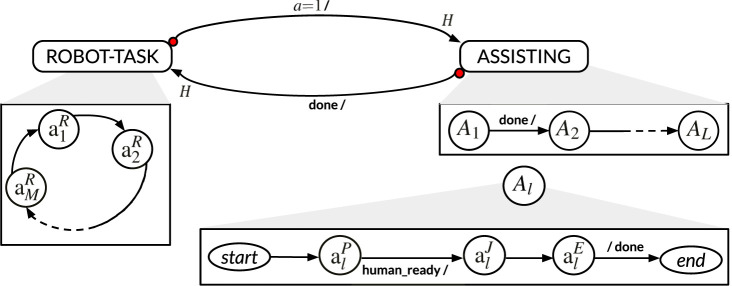
Hierarchical state machine depicting the collaborative task from the robot perspective. The robot transitions from *ROBOT-TASK* to *ASSISTING* in between states $a_{i}^{R}$ if a=1 . Once an (*assist*) action $A_{i}$ is completed, the robot goes back to its task. The state machine follows the conventions as in Lee and Seshia (2017). Each transition is labeled with *guard*/*effect*. The *guard* determines whether the transition may be taken on a reaction. The *effect* specifies what outputs are produced on each reaction. *H* denotes a *history* transition. The red dot a *preemptive* transition.

To assist the human, the robot must first complete its current robot-task action $a_{i}^{R}$ before pausing its ongoing task. Once paused, the robot performs a *preparatory* action (e.g., repositioning or collecting a tool) to prepare for the joint action. After completing the joint action, the robot executes a *homing* action before either resuming its task or preparing for the next joint action. The sets of preparatory and homing actions are denoted as ${\left\{a_{l}^{P}\right\}}_{l=1}^{L}$ and ${\left\{a_{l}^{E}\right\}}_{l=1}^{L}$ , respectively. A depiction of the task as a hierarchical state machine is provided in Figure 3.

Additionally, we assume access to a set of Q human demonstrations for each *non-joint* human action $a_{j}^{H}\in H\backslash J$ . These trajectories, denoted as $Y_{j}={\left\{y_{k}^{j}\right\}}_{k=1}^{Q}$ , consist of the Cartesian positions of the human hand along the x -, y -, and z -axes.

The objective is to minimize the total idle times for both the robot $\left(\Delta_{\text{total idle}}^{R}\right)$ and the human $\left(\Delta_{\text{total idle}}^{H}\right)$ . This is achieved by optimizing the cost function:

$$C\left(\Delta_{\text{total idle}}^{R},\Delta_{\text{total idle}}^{H}\right):=\Delta_{\text{total idle}}^{R}+\lambda \Delta_{\text{total idle}}^{H},$$ (2)

where λ>0 is a weighting coefficient that balances their relative importance.

#### 2.4.2 POMDP formulation

The collaboration problem is modeled as a finite-horizon episodic POMDP. In this framework, the robot acts as an agent that makes binary decisions between robot-task actions–whether to assist the human or continue its task–while the human is treated as part of the environment. Formally, the POMDP is defined by the tuple (S,A,T,R,Ω,O) , where:

• S is the state space;
• A={0,1} is the binary set of *policy* actions;
• T:S×A×S→[0,1] is the transition probability;
• R:S×A×S→R is the reward function;
• Ω is the observation space;
• O:S→Ω is the observation function.

Note that *policy* actions a∈A should not be confused with the *task* actions $\left(a_{i}^{R},a_{l}^{P},a_{l}^{J},a_{l}^{E}\right)$ defined in the previous section.

Each element of the state space S is defined as $s=\left(a_{i}^{R},a_{j}^{H},a_{l}^{J},\Delta_{\text{start}}^{H},y^{H},\Delta_{\text{idle}}^{R},\Delta_{\text{idle}}^{H}\right)$ , where:

-
$a_{i}^{R}\in R$ is the last robot-task action;
-
$a_{j}^{H}\in H$ is the current human action;
-
$a_{l}^{J}\in J$ represents the joint action that human and robot should perform next;
-
$\Delta_{\text{start}}^{H}\geq 0$ is the elapsed time since the start of $a_{j}^{H}$ ;
-
$y^{H}$ is the observed human hand trajectory during $a_{j}^{H}$ ;
-
$\Delta_{\text{idle}}^{R},\Delta_{\text{idle}}^{H}\geq 0$ are the idle times observed during the last transition for the robot and human, respectively.

The transition function $T\left(s,a,s^{\prime }\right):=P\left(s^{\prime }|a,s\right)$ is the probability of the state evolving from s to $s^{\prime }=\left(a_{i^{\prime }}^{R},a_{j^{\prime }}^{H},a_{l^{\prime }}^{J},\Delta_{\mathrm{start}}^{\prime H},y^{\prime H},\Delta_{\mathrm{idle}}^{\prime R},\Delta_{\mathrm{idle}}^{\prime H}\right)$ . The state variables evolve as follows:

$$a_{i^{\prime }}^{R}=\left\{\begin{array}{cc} a_{\left(i+1\right)\;\mathrm{mod}\;M}^{R}\; & a=0\\ a_{i}^{R}\; & a=1 \end{array}\right.$$

$$a_{l^{\prime }}^{J}=\left\{\begin{array}{cc} a_{l}^{J}\; & a=0\\ a_{l+1}^{J}\; & a=1, \end{array}\right.$$

with remaining state variables $\left(\Delta_{\text{start}}^{\prime H},y^{\prime H},\Delta_{\text{idle}}^{\prime R},\Delta_{\text{idle}}^{\prime H}\right)$ updated based on observed interactions. In the next section we describe a model to simulate the evolutions of these quantities.

The reward directly minimizes the cost defined in Equation 2:

$$R\left(s,a,s^{\prime }\right):=-\Delta_{\text{idle}}^{R}-\lambda \Delta_{\text{idle}}^{H}.$$

The observation function is defined as $O\left(s\right):=\left(a_{i}^{R},a_{j}^{H},\Delta_{\text{start}}^{H},\tau^{H}\right)$ , where $\tau^{H}=\phi_{y^{H}}\left(\Delta_{\mathrm{start}}^{H}\right)\in \left[0,1\right]$ represents the estimated completion percentage of $a_{j}^{H}$ , computed from $y^{H}$ using OS-DTW_WP_. For each human-only action $a_{j}^{H}$ , we select the first trajectory $y_{1}^{j}\in Y_{j}$ in the training set as the reference for OS-DTW_WP_. Thus, the observation consists of the last robot-task action, current human action, elapsed time, and phase estimate. The definition of the observation space Ω follows accordingly.

#### 2.4.3 Simulated environment

Training an RL policy directly on physical hardware is impractical due to time constraints and the constant requirement of human involvement in the task. To address this, we develop a simulated environment that models the collaborative task defined in Section 2.4.1, leveraging human demonstrations to approximate real-world dynamics. This environment enables efficient training of online and on-policy RL algorithms while preserving the POMDP structure formalized in Section 2.4.2.

To model the collaborative task, we assume the duration of each action follows a Gaussian distribution, and estimate them from demonstration data. Specifically, $\Delta_{k}^{X}∼N\left(\mu_{X_{k}},\sigma_{X_{k}}^{2}\right)$ , where X∈{H,R,P,E} corresponds to human, robot-task, preparatory, and homing actions, respectively.

At the beginning of each episode, we sample from these distributions the durations human actions ${\left\{\Delta_{j}^{H}\right\}}_{j=1}^{N}$ , preparatory actions ${\left\{\Delta_{l}^{P}\right\}}_{l=1}^{L}$ , and homing actions ${\left\{\Delta_{l}^{E}\right\}}_{l=1}^{L}$ . Then, one trajectory ${\tilde{y}}_{j}$ is sampled from the set of demonstrations $Y_{j}$ for each *non-joint* action $a_{j}^{H}$ .

Moreover, to avoid overfitting on the training data, we linearly rescale the time axis of each trajectory ${\tilde{y}}_{j}$ to align with each sampled duration $\Delta_{j}^{H}$ . As a result, each new trajectory represents either a compressed or stretched version of an actual demonstration. We found this augmentation essential for ensuring robustness and improving the policy’s generalization capabilities.

By employing these quantities, in addition to the state evolution dynamics described in Section 2.4.2, we model the transitions of the POMDP from $s=\left(a_{i}^{R},a_{j}^{H},a_{l}^{J},\Delta_{\text{start}}^{H},y^{H},\Delta_{\text{idle}}^{R},\Delta_{\text{idle}}^{H}\right)$ to $s^{\prime }\;=\;\left(a_{i^{\prime }}^{R},a_{j^{\prime }}^{H},a_{l^{\prime }}^{J},\Delta_{\mathrm{start}}^{\prime H},y^{\prime H},\Delta_{\mathrm{idle}}^{\prime R},\Delta_{\mathrm{idle}}^{\prime H}\right)$ as:

$$a_{j^{\prime }}^{H}=\beta_{\left(a_{j}^{H},\Delta_{\mathrm{start}}^{H}\right)}\left(\Delta \right)$$

$$\Delta_{\mathrm{start}}^{\prime H}=\Delta -\Delta_{\mathrm{start}}^{H}-\overset{j^{\prime }-1}{\underset{k=j}{\sum }}\Delta_{k}^{H}$$

$$y^{\prime H}={\tilde{y}}_{j^{\prime }}\left(0:\Delta_{\mathrm{start}}^{\prime H}\right)$$

$$\Delta_{\mathrm{idle}}^{\prime R}=\left\{\begin{array}{cc} 0\; & a=0\\ \max\left\{0,\overset{\alpha \left(l\right)-1}{\underset{k=j}{\sum }}\;\Delta_{k}^{H}-\Delta_{\mathrm{start}}^{H}-\Delta_{l}^{P}\right\}\; & a=1 \end{array}\right.$$

$$\Delta_{\mathrm{idle}}^{\prime H}=\left\{\begin{array}{cc} \max\left\{0,\Delta^{R}+\Delta_{\mathrm{start}}^{H}-\overset{\alpha \left(l\right)-1}{\underset{k=j}{\sum }}\;\Delta_{k}^{H}\right\}\; & a=0\\ \max\left\{0,\Delta_{l}^{P}+\Delta_{\mathrm{start}}^{H}-\overset{\alpha \left(l\right)-1}{\underset{k=j}{\sum }}\;\Delta_{k}^{H}\right\}\; & a=1 \end{array}\right.$$

$\Delta^{R}∼N\left(\mu_{R_{i^{\prime }}},\sigma_{R_{i^{\prime }}}^{2}\right)$ is the duration of the robot-task $a_{i^{\prime }}^{R}$ .

Δ is the duration of the transition:

$$\Delta =\left\{\begin{array}{cc} \Delta^{R}\; & a=0\\ \Delta_{l}^{P}+\Delta_{\alpha \left(l\right)}^{H}+\Delta_{l}^{E}\; & a=1. \end{array}\right.$$

β is a function that, given the current human action $a_{j}^{H}$ and its elapsed time $\Delta_{\mathrm{start}}^{H}$ , returns the ongoing human action after a time Δ , namely,

$$\beta_{\left(a_{j}^{H},\Delta_{\mathrm{start}}^{H}\right)}\left(\Delta \right):=\underset{{\text{a}}_{j^{\prime }}^{H}}{\mathrm{arg min}}\left\{j^{\prime }\geq j\;|\;\Delta \leq \overset{j^{\prime }}{\underset{k=j}{\sum }}\Delta_{k}^{H}\right\}.$$

Moreover we assume that the human always starts from the first action, while the robot is already in operation. As a result, the initial state is non-deterministic. Specifically, we assume the first human action to start when the robot is performing an action $a_{i}^{R}$ .

We derive the initial robot action by sampling from a generalized Bernoulli distribution $D^{R}$ . Namely, each action $a_{i}^{R}\in R$ has a probability:

$$P\left(a_{i}^{R}\right)=\frac{\mu_{R_{i}}}{\sum_{k=1}^{M}\mu_{R_{k}}}.$$

Then, initialize the state as:

$$a_{l}^{J}=a_{1}^{J}$$

$$a_{i}^{R}∼D^{R}$$

$$\Delta_{\mathrm{start}}^{H}=U\left[0,N\left(\mu_{R_{i}},\sigma_{R_{i}}^{2}\right)\right]$$

$$a_{j}^{H}=\beta_{\left(a_{1}^{H},0\right)}\left(\Delta_{\mathrm{start}}^{H}\right)$$

$$y^{H}={\tilde{y}}_{j}\left(0:\Delta_{\mathrm{start}}^{H}\right)$$

$$\Delta_{\mathrm{idle}}^{R}=0$$

$$\Delta_{\mathrm{idle}}^{H}=\max\left\{0,\Delta_{\mathrm{start}}^{H}-\overset{\alpha \left(l\right)}{\underset{k=1}{\sum }}\Delta_{k}^{H}\right\},$$

where U denotes the uniform distribution.

#### 2.4.4 RL algorithm: proximal policy optimization

To solve the POMDP described in Section 2.4.2 within the simulated environment of Section 2.4.3, we select the Proximal Policy Optimization (PPO) (Schulman et al., 2017) algorithm. Our choice is motivated by four key factors:

- *Hybrid State Space Handling*: PPO natively supports state spaces combining both discrete $\left(a_{i}^{R},a_{j}^{H}\right)$ and continuous observations $\left(\Delta_{\text{start}}^{H},\tau^{H}\right)$ , eliminating the need for algorithm modifications.
- *Stability*: PPO employs a clipped surrogate objective that constrains policy updates, which prevents large variations of the policy and ensures learning stability despite noisy phase estimates and incomplete state observations. This is particularly important in our framework, as human behavior is highly stochastic and unpredictable.
- *Variance Handling*: On-policy advantage estimation accounts for actual interaction variance, critical given the stochasticity of human action durations.
- *Efficiency*: PPO achieves superior sample efficiency which is critical for human-robot collaboration where data collection is costly.

While alternative algorithms could be considered, PPO avoids several pitfalls that make them less suitable for our application. For example, Deep Q-Networks (DQN) (Huang, 2020) are limited to discrete action spaces and would require explicit discretization of continuous inputs, while also struggling with partial observability. Trust Region Policy Optimization (TRPO) (Schulman et al., 2015) shares many of PPO’s theoretical guarantees, yet, it incurs significantly higher computational overhead without providing empirical gains for binary action spaces. In the case of Soft Actor-Critic (SAC) (Masadeh et al., 2019), its design for continuous control introduces unnecessary complexity when applied to discrete action spaces. Overall, PPO emerges as the most suited choice for the PACE framework, striking an optimal balance between simplicity, sample efficiency, and performance.

## 3 Experiments

**FIGURE 4:**
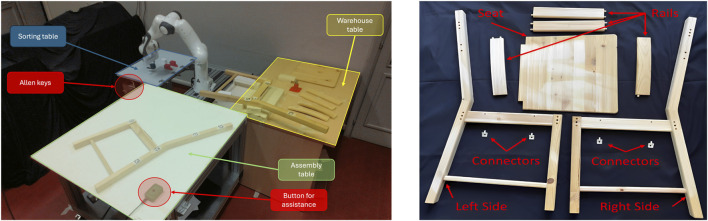
Experimental setup for the wooden chair assembly process. On the left, the robotic workcell andworking areas, including tables for specific parts of the process (sorting, warehouse, assembly). On the right, the disassembled components of the IKEA Ivar chair, including: the left and right sides of the chair,and the rails connecting these sides.

We evaluated the proposed methods in a real-world scenario through a pilot study involving the assembly of an IKEA chair (see Fig. Figure 4). We collected human hand trajectories from users performing a collaborative assembly task with a robot manipulator. This task also serves to test the PACE framework, both demonstrating a practical application of OS-DTW_WP_ and showcasing the efficacy of PACE in enhancing human-robot synergy.

### 3.1 Experimental setup

The experimental setup consists of the robotic workcell shown in Figure 4, including a Franka Emika Panda manipulator and three main working areas: a *sorting table* for the robot task, a *warehouse table* where the components to be assembled are stored, and an *assembly table* where the collaborative assembly process takes place. The chair is the IKEA Ivar wooden chair^1^ reported in Figure 4, where the dowel pins have been substituted with neodymium magnets to simplify the rails insertion step. The robot is programmed using the ROS^2^ and MoveIt ]^3^ frameworks. An RGB-D camera monitors the area around each table, utilizing April-Tag (Malyuta et al., 2019) markers to locate the chair components. Participants were equipped with the Xsens MVN Awinda motion capture system (Schepers et al., 2018), which recorded the position of their right hand at a sampling rate of 10 Hz. Alternative tracking gloves such as Rokoko^4^, HaptX^5^, could be employed.

### 3.2 Task description

A typical chair assembly process involves several steps, including positioning the chair sides, placing the rails, aligning the screws, and tightening the components together.

In our setup, the human operator performs the majority of the assembly but requires the robot’s assistance at specific stages, such as transporting large components or handing over tools. Meanwhile, the robot concurrently carries out an independent task involving a series of cube sorting operations.

As shown in Figure 4, the chair assembly process begins with the right side of the chair already positioned on the *assembly table*, and the remaining chair components on the *warehouse table*. According to the formulation described in 2.4.1, the process is outlined as follows:

1. The human connects the 4 rails to the right side of the chair. This is denoted as *rail placing* action, which corresponds to $a_{1}^{H}$ .
2. The robot and human collaboratively transport the left side from the warehouse area and place it on top of the rails $\left(a_{1}^{J}\right)$ .
3. The human adjusts the chair side and places 3 screws on top. This is the *screw placing* action and corresponds to $a_{3}^{H}$ .
4. The robot hands an Allen key to the human $\left(a_{2}^{J}\right)$ .
5. The human uses the key to tighten two of the screws. This is the *screwing* action, corresponding to $a_{5}^{H}$ .
6. The robot hands over a second Allen key to allow the human to tighten the remaining screw $\left(a_{3}^{J}\right)$ .

**FIGURE 5:**
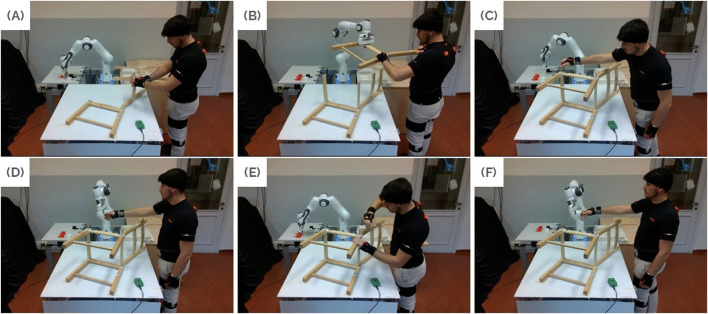
Main steps in the collaborative assembly process. **(A)** *Rail placing*. **(B)** Collaborative transport of the left side of the chair. **(C)** *Screw placing*. **(D)** Handover of the first Allen key. **(E)** *Screwing*. **(F)** Handover of the second Allen key to tighten the last screw. Figure contains images of the author(s) only.

A depiction of the collaborative assembly process considered in the pilot study is reported in Figure 5, highlighting the main steps described above.

### 3.3 Data collection

To gather the training trajectories, we employed a system where users explicitly requested assistance from the robot via a button press. We collected data from 5 subjects, with each subject performing the experiment 4 times. Additionally, one of the subjects provided an extra demonstration to generate the references for the DTW algorithms. Thus, we employed a total of 21 trajectories per action to tune or train the various methods.

For reference, the average duration of each human action $a_{i}^{H}$ was approximately 22 s for rail placing, 18 s for screw placing, and 40 s for screwing. The robot preparatory actions $a_{i}^{P}$ for the following joint actions took on average 11 s, 8 s, and 9 s, respectively. Each robot cube sorting move, represented by the action $a_{i}^{R}$ , had a duration of approximately 8 s.

### 3.4 PACE training

We implemented the POMDP described in 2.4.2 as a custom Gymnasium environment (Towers et al., 2024) and used the Stable-Baselines3 library (Raffin et al., 2021) for training the PACE policy. Out of the 4 demonstrations per subject, 3 were used for training and 1 for validation. Moreover, based on Decker et al. (2017); Brosque and Fischer (2022), in our experiments we assume that the cost of employing a robot is approximately one-third of the cost of human labor, thus setting the parameter λ of the reward function equal to 3.

### 3.5 Test experiments design

The test experiments involved 12 volunteers (5 women and 7 men) aged 24 to 28, two of whom also participated as training subjects.

In addition to the PACE framework, which monitors human task progression, participants tested two alternative systems. The first is a baseline system (*explicit query*) where the human operator explicitly requests robot assistance via a button after completing each action. The second is a variant of the PACE framework (*PACE w/o phase*), which operates without actively monitoring users or using phase information.

Participants received instructions on the assembly task and the robot’s action capabilities. Each participant tested all three systems (*explicit query*, *PACE w/o phase*, and *PACE*) in a randomized order, completing two trials for each system. No prior information was provided to the users regarding the differences between the two proactive policies. After each set of trials, participants filled out two surveys: the NASA-TLX (Hart and Staveland, 1988), and a custom 5-point Likert scale questionnaire (see Figure 12).

From the experiments conducted with the *PACE* framework, we collected a total of 24 trajectories per action from different subjects, which were used as the test dataset to validate our methods.

## 4 Results

**FIGURE 6:**
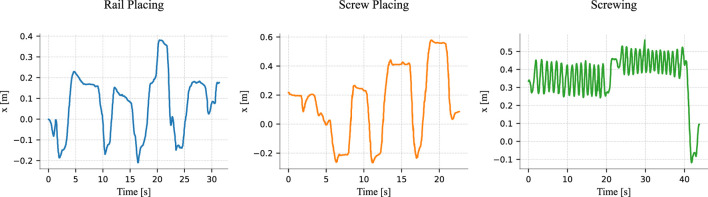
Reference trajectories for the rail placing, screw placing, and screwing tasks. The four peaks in the rail placing trajectory correspond to the placement of each rail. Similarly, the screw placing trajectory exhibits three peaks, each representing the placement of a screw, but it also includes an initial transient where the human adjusts the position of the chair side. The screwing trajectory corresponds to the tightening of two different screws with an Allen key, where each small oscillation matches one turn of the key.

As outlined in the previous section, all the results presented hereafter are derived from a dataset consisting of 24 trajectories per action. The training dataset includes 20 trajectories per action, along with one reference trajectory. The reference trajectories are reported in Figure 6.

### 4.1 Phase estimation

As discussed in Section 2.2.2, defining a ground truth for the phase estimate is not straightforward. The alignment produced by Dynamic Time Warping (DTW) depends on the chosen distance metric and constraints, and different metrics or constraints can yield varying alignments. Since there is no universal rule for selecting the “best” metric or constraints, this introduces subjectivity and makes it challenging to establish an objective ground truth. While human annotations could be used to define alignments based on interpretation, they are inherently subjective, inconsistent, and thus unreliable as a ground truth.

**FIGURE 7:**
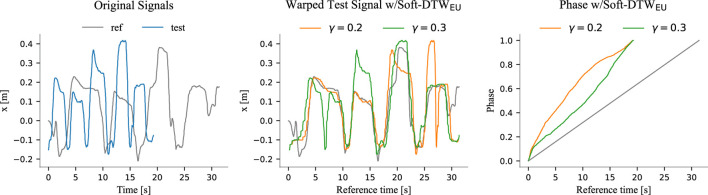
The left plot shows the x-dimensions of the original reference and test trajectories. The middle plot displays the aligned test trajectory using Soft-DTW_EU_ for two different parameters γ , and the corresponding phases are shown in the right plot, where the gray line represents the reference phase evolution. By examining the original signals, we observe that the test trajectory is shorter than the reference trajectory, indicating that the user performed the action faster. This is reflected in the phase plot, where the phases of the test trajectory exhibit a steeper slope compared to the reference phase evolution. These plots refer to a rail-placing experiment, where each spike ideally represents the placement of one rail by the user. However, the test trajectory presents five spikes due to a blunder: one of the rails fell and needed to be repositioned. Soft-DTW_EU_ aligns the first two spikes together for γ=0.3 , while it aligns the last two spikes for γ=0.2 . Nevertheless, there is no clear optimal choice between the two alignments.

**FIGURE 8:**
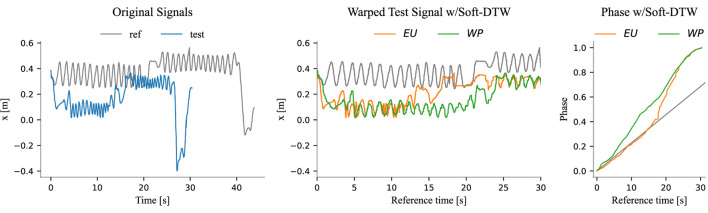
These plots are similar to those shown in Figure 7 but correspond to a screwing experiment. Here, we highlight the differences between Soft-DTW_EU_ and Soft-DTW_WP_. The middle plot has been truncated for better visualization. Each oscillation in the signal ideally represents one turn of the screw. By looking at the middle plot, it is evident that the method relying on the Euclidean distance fails to align the signals effectively.

For these reasons, in the following results, we employ Soft-DTW—as in Cuturi and Blondel (2017)—to obtain the ground truth phases where applicable^6^. All trajectories were manually inspected to determine the optimal parameter γ for Soft-DTW. However, in some cases, no clear optimum exists, and multiple plausible ground truths may emerge. An example of this is illustrated in Figure 7. Additionally, we observed that Soft-DTW struggled to align many trajectories in the screwing task effectively, as illustrated in Figure 8. As discussed in Section 2.2.1, the Euclidean distance bases its alignment on the absolute value of the signals, making it less effective at capturing local patterns—a task in which the Windowed-Pearson (WP) distance excels. Therefore, for the screwing experiments, we adopted a modified version of Soft-DTW that utilizes the WP distance, referred to as Soft-DTW_WP_. To clarify the distinction, we refer to the “standard” Soft-DTW as Soft-DTW_EU_, explicitly indicating its reliance on the Euclidean distance.

**FIGURE 9:**
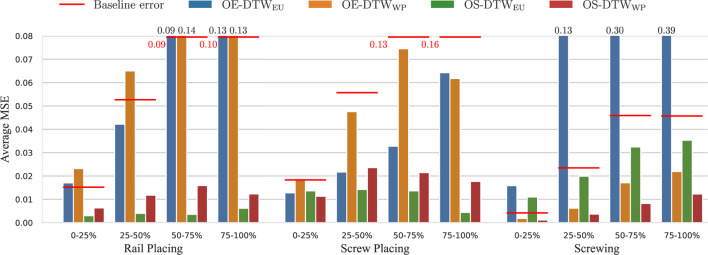
Average mean squared error (MSE) of the estimated phase with respect to the ground truth computed with Soft-DTW^6^. The bar plot shows the results across the three tasks (rail placing, screw placing, and screwing) for OE-DTW_EU_, OE-DTW_WP_, OS-DTW_EU_, and OS-DTW_WP_. Results are reported over the 1st, 2nd, 3rd, and 4th quarters of the trajectories. Values of the bars exceeding the vertical limit are indicated on top.

Figure 9 shows the average Mean Square Errors (MSE) of the estimated phase for Open-end DTW and Open-end Soft-DTW, using either the Euclidean distance or the Windowed-Pearson distance. All parameters ( γ , w ) where tuned separately for each method and task to minimize the average MSE across the entire trajectories. The bar plot shows the errors divided into quartiles: the average error is calculated for each quarter of the trajectories (0%–25%, 25%–50%, 50%–75%, and 75%–100%). The baseline error is the error obtained by considering a nominal phase evolution, i.e.:

$$\phi_{\text{nom}}\left(t\right)=\min\left\{1,\frac{t}{\;\bar{t}\;}\right\},$$

where $\bar{t}$ is the *nominal duration* as in Section 2.3.1.

We observe that the baseline error increases with each quartile, as expected. This trend occurs because, similar to the reference phases depicted in Figures 7, 8, the estimated phases progressively deviate from the true phase over time. This deviation is a natural consequence of the linear approximation used to model phase evolution, where errors accumulate as the trajectory progresses. The longer the trajectory, the larger the error tend to become. While the same reasoning applies to DTW-based methods, the availability of more data enables the algorithms to refine the alignments dynamically. This results in non-monotonic error trends, as improved alignment with additional data can mitigate error growth. Especially in rail placing and screw placing, OE-DTW_WP_ and OS-DTW_EU_ demonstrate their ability to better align trajectories as more data becomes available, effectively reducing errors even as sequences lengthen.

From these results we notice that classical Open-end DTW (OE-DTW_EU_) performs poorly in all tasks. The same applies to its version incorporating the WP distance (OE-DTW_WP_) with the exception of the screwing task, where OE-DTW_WP_ performs even better than OS-DTW_EU_, namely the soft DTW employing the Euclidean distance.

OS-DTW_EU_ achieves the best performance in the two placing tasks, yet it performs poorly in screwing. The Open-End Soft-DTW with the WP distance (OS-DTW_WP_) excels in screwing, where the other methods struggle, while maintaining good performances in the other two.

In rail placing and screw placing, the Euclidean distance proves effective because absolute rail and screw positions remain consistent across trials. In the screw placing task, the initial transient phase–where users adjust the chair side (as described in Section 3.2 and illustrated in Figure 6)–introduces unstructured local hand motions. These variations are sometimes misinterpreted by the WP distance due to its reliance on local window correlations. In contrast, the Euclidean distance succeeds by focusing on global hand-position consistency, which remains relatively stable during adjustments. Nevertheless, in the screwing task, Euclidean metrics struggle with divergent absolute screwdriver positions, while the WP distance thrives by matching local rotational patterns, such as the repetitive turns of the Allen key.

These findings highlight a critical insight: while the use of the soft minimum mitigates temporal misalignment, the choice of distance metric determines whether global positional trends or local shape similarities drive the alignment. This decision must align with the dominant features of the target task, emphasizing the importance of selecting the method that better aligns with the specific characteristics of the application.

### 4.2 Action completion time estimation

As discussed in Section 2.3.2, we expected the linear method to improve over time and, on average, surpass the nominal method after a certain elapsed time or time percentage, once sufficient data were available to estimate the phase and the user’s execution speed accurately. For these reasons, and to enhance the robustness of the hybrid method against potential distribution shifts, we employed both switching criteria jointly. Specifically, in the hybrid method, the linear method replaces the nominal method only when both conditions are met: t>t* and τ>τ* .

**FIGURE 10:**
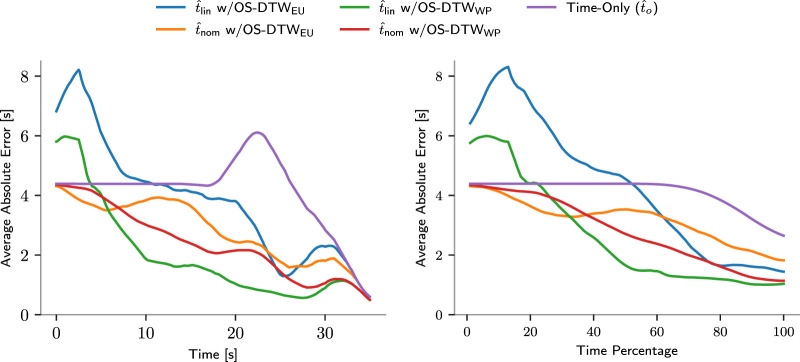
Average Absolute Errors for Rail Placing. The left plot depicts the average error over time, while the right plot shows it with respect to the percentage of the total duration of each trajectory. Curves have been smoothed for clarity.

A significant advantage of switching to the linear method over the nominal one was observed in the training data only for OS-DTW_WP_ in the rail placing and screwing tasks. The results relative to the rail placing training experiments are shown in Figure 10.

All results on the test datasets are summarized and reported in Table 1.

**TABLE 1:** Average Mean Absolute Error (seconds) per quartile (Q1: 0%–25%, Q2: 25%–50%, Q3: 50%–75%, Q4: 75%–100%) for each task.

Phase method	Estimation method	Rail placing				Screw placing				Screwing			
		Q1	Q2	Q3	Q4	Q1	Q2	Q3	Q4	Q1	Q2	Q3	Q4
OE-DTW_EU_	Nominal	4.14	3.54	4.59	6.14	4.71	4.10	3.43	3.91	7.78	15.44	22.48	24.87
	Linear	38.25	27.01	29.87	16.05	64.11	9.55	7.53	7.75	201.7	544.5	419.2	322.2
OE-DTW_WP_	Nominal	4.81	5.25	6.68	6.92	5.34	6.05	5.55	4.68	6.71	5.33	4.72	3.69
	Linear	120.9	11.79	14.88	10.16	113.6	21.83	11.56	7.20	24.28	5.56	5.31	4.29
OS-DTW_EU_	Nominal	**2.79**	**1.95**	**1.73**	**1.37**	**3.55**	**2.92**	**1.62**	1.05	7.03	6.49	7.18	5.65
	Linear	7.86	5.25	3.18	1.46	7.91	3.65	2.08	**0.99**	18.44	10.01	9.93	6.96
	Hybrid	**2.79**	**1.95**	**1.73**	**1.37**	**3.55**	**2.92**	**1.62**	1.05	7.03	6.49	7.18	5.67
OS-DTW_WP_	Nominal	3.62	2.58	2.42	1.79	4.88	4.72	3.12	1.95	**6.39**	5.30	4.24	**2.95**
	Linear	5.62	3.48	2.87	1.62	7.87	5.14	2.53	1.63	13.18	4.04	4.07	3.33
	Hybrid	3.56	3.52	3.09	1.82	4.88	4.75	2.97	1.96	6.49	**4.01**	**3.80**	3.19
-	Time-Only	4.02	4.02	4.02	3.98	4.82	4.82	4.82	4.46	6.97	6.97	6.97	5.86

Values exceeding those obtained with the Time-Only $\left({\hat{t}}_{o}\right)$ method are shown in red. The minimum values for each task and quartile are highlighted in bold.

As demonstrated in the phase results section, the “non-soft” DTW methods generally perform poorly, leading to larger estimation errors when using the linear time estimation method.

For the soft DTW methods, switching from the nominal to the linear method is beneficial in approximately half of the cases. The nominal and hybrid methods using OS-DTW_EU_ were the best in the rail placing and screw placing tasks. In contrast, the hybrid method using OS-DTW_WP_ was the overall best in the screwing task.

Overall, the hybrid method either outperformed or matched the performance of the other methods in each task. However, it did not achieve ideal results in the rail placing and screw placing tasks with OS-DTW_WP_, likely due to variations in the optimal switching time and phase between the training and test datasets.

### 4.3 Collaborative assembly results

The goal of this experiment is to evaluate whether introducing proactive robot behavior can reduce downtime during assembly and enhance the quality of the assembly experience from the user’s perspective. Additionally, we aim to demonstrate that monitoring human progress—specifically using OS-DTW_WP_—can further improve human-robot synergy, both in terms of reducing waiting times and enhancing user experience.

To this end, as explained in Section 3.5, we tested three different systems (*explicit query*, *PACE w/o phase*, and *PACE*) in real-user experiments. *PACE* employs OS-DTW_WP_ to estimate the phase.

**FIGURE 11:**
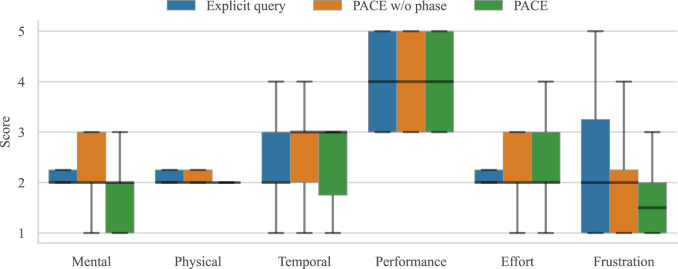
NASA-TLX (Hart and Staveland, 1988) findings for subjective measures on a 5-point scale ranging from *Very Low*to *Very High*. The boxplot displays medians, interquartile ranges, and whiskers representing the data distribution. The questions are the following. Mental: *How mentally demanding was the task?*Physical: *How physically demanding was the task?*Temporal: *How hurried or rushed was the pace of the task?*Performance: *How successful were you in accomplishing the task?*Effort: *How hard did you have to work to accomplish this task?*Frustration: *How insecure, discouraged, irritated, stressed, or annoyed were you?*

**FIGURE 12:**
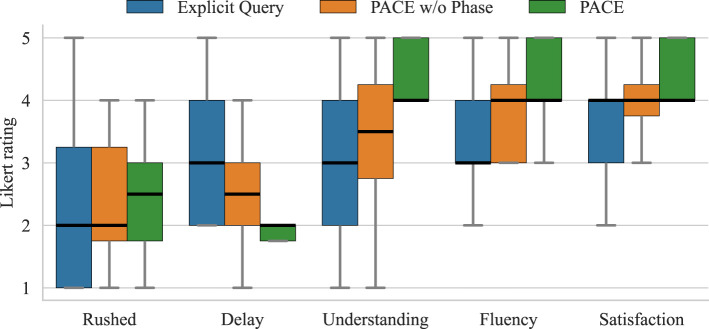
Findings for subjective measures on a 5-point scale ranging from *Strongly Disagree*to *Strongly Agree*. The boxplot displays medians, interquartile ranges, and whiskers representing the data distribution. The questions are the following. Rushed: *I felt rushed by the robot’s actions*. Delay: *I felt that the robot took too long to assist me*. Understanding: *I felt the robot had a good understanding of the task*. Fluency: *The robot and I collaborated fluently*. Satisfaction: *I feel satisfied by the performance of the system*.

To assess subjective aspects, we employed both the NASA Task Load Index (Hart and Staveland, 1988), a widely used multidimensional assessment tool that rates perceived workload (see Figure 11), and a custom 5-point Likert scale questionnaire (see Figure 12) specifically tailored for the task at hand.

For each experiment, we recorded the robot idle times and a video of the entire assembly. The video recordings were later inspected to annotate the precise end of each human action to calculate the relative waiting times. We report the quantitative results of the robot’s and users’ waiting times before each of three joint action in Table 2.

**TABLE 2:** Collaborative assembly results of real experiments (12 subjects, 2 trials per method). Columns include human and robot waiting times for A1 (Rail Placing), A2 (Screw Placing), and A3 (Screwing) tasks, and overall cost. The value in bold indicates the lowest cost.

Real experimental results									
System	Robot Waiting Time [s]				Human Waiting Time [s]				Cost
	A1	A2	A3	Total	A1	A2	A3	Total	(λ=3)
Explicit query	0.0	0.0	0.0	0.0	14.00	11.28	12.15	37.43	112.3
PACE w/o phase	1.56	3.48	11.64	16.68	1.79	1.02	1.06	3.87	28.29
PACE	1.93	2.65	1.28	5.86	1.20	0.56	2.56	4.32	**18.81**

As expected, the system with the explicit query appears to be the most penalizing for the user’s waiting time. This is also reflected in the subjective user results, which generally indicated that the robot took too long to provide assistance. Excluding the baseline—which always shows null robot idle time by design—Table 2 demonstrates that the method employing the phase estimate reduces robot idle time nearly to one-third, without significantly impacting the user’s waiting time. Moreover, as shown in Figure 12, the method that monitors human progress outperforms the others in subjective measures as well. The users reported a higher level of fluency, understanding, and overall satisfaction, confirming that the method adapts to the pace of various participants. Additionally, Figure 11 shows that a proactive robot operating autonomously does not negatively impact mental strain or the overall Task Load Index. Notably, five out of twelve participants explicitly stated in the open comment section of the questionnaire that they preferred the system monitoring human task advancement, with many appreciating that it provided assistance only as they neared the end of their action.

Moreover, in Table 3, we report the results obtained with the training data on the POMDP defined in Section 2.4.2. The *PACE w/EU* system reported in the tables, refers to a variant of the PACE policy that employs OS-DTW_EU_ to estimate the phase. The *oracle* system represents an ideal policy with posterior knowledge of the completion time of each action, serving as a theoretical lower bound.

**TABLE 3:** Collaborative assembly results obtained in simulation with the real training trajectories (5 subjects, 4 trajectories per subject and method). Results are averaged across 1000 different seeds. Columns include human and robot waiting times for A1 (Rail Placing), A2 (Screw Placing), and A3 (Screwing) tasks, and overall cost. The value in bold indicates the lowest cost achieved without hindsight information.

Training trajectories									
System	Robot Waiting Time [s]				Human Waiting Time [s]				Cost
	A1	A2	A3	Total	A1	A2	A3	Total	(λ=3)
Explicit query	0.0	0.0	0.0	0.0	14.13	11.27	11.95	37.35	112.05
PACE w/o phase	4.10	5.99	12.49	22.57	0.58	0.07	1.11	1.76	27.87
PACE w/EU	4.20	5.42	13.89	23.51	0.43	0.17	1.08	1.69	28.59
PACE	1.90	6.10	5.45	13.45	0.92	0.10	0.81	1.83	**18.94**
Oracle	1.96	1.32	1.83	5.11	0.25	0.26	0.28	0.79	7.49

For a fairer comparison, we replayed the test trajectories using the same POMDP and report the results in Table 4. Quantitatively, we observe minimal benefits from phase estimation methods (including the oracle) in both rail-placing and screw-placing actions. We attribute this to the relatively short duration of these human actions compared to robot preparation and task execution times, as the combination of lengthy robot operations with brief human actions fundamentally limits the potential advantages of progress estimation in this context. While PACE outperforms all baselines, PACE w/EU fails to improve over PACE w/out phase. This aligns with OS-DTW_EU_’s poor performance during screwing actions.

**TABLE 4:** Collaborative assembly results obtained in simulation by replaying the test trajectories. Results are averaged across 1000 different seeds. Columns include human and robot waiting times for A1 (Rail Placing), A2 (Screw Placing), and A3 (Screwing) tasks, and overall cost. The value in bold indicates the lowest cost achieved without hindsight information.

Test trajectories replayed									
System	Robot Waiting Time [s]				Human Waiting Time [s]				Cost
	A1	A2	A3	Total	A1	A2	A3	Total	(λ=3)
PACE w/o phase	1.67	2.91	10.90	15.48	1.37	0.52	0.68	2.58	23.22
PACE w/EU	2.09	2.59	11.12	15.79	0.99	0.62	0.61	2.23	22.40
PACE	1.96	2.77	1.27	5.99	1.09	0.57	2.58	4.25	**18.75**
Oracle	2.19	1.16	1.56	4.91	0.25	0.54	0.28	1.07	8.12

## 5 Discussion

Our experimental results demonstrate that real-time progress estimation, enabled by novel Open-end Soft-DTW variants, addresses critical gaps in human-robot collaboration (HRC) systems. While most existing methods reactively monitor human activity at the task level (Cheng et al., 2021; Ramachandruni et al., 2023), they lack mechanisms to accommodate the natural variability in human execution pace. In contrast, our approach monitors individual actions at the atomic level, enabling robots to dynamically synchronize with their human counterparts–a capability missing from previous frameworks.

Early attempts to estimate progress, such as Open-end DTW (Maderna et al., 2019), introduced temporal flexibility but proved oversensitive to real-world trajectory variations. Our work bridges this gap with Open-end Soft-DTW (OS-DTW_EU_), which mitigates local minima through a softmin operator, marking its first real-time application in HRC. This innovation allows robust handling of unpredictable human motions while maintaining computational feasibility.

The task-dependent performance of our methods highlights the need for context-aware estimation. For instance, OS-DTW_WP_ excelled in screwing actions by capturing local rotational patterns, whereas OS-DTW_EU_ proved superior in placement tasks, where positional consistency is paramount. These findings suggest that developing new distance metrics, combining existing ones, or selecting metrics based on training data or task semantics could further enhance adaptability.

The practical impact of these advancements is evident in the PACE framework, which reduced robot idle times by nearly two-thirds while maintaining low user waiting times. Unlike reactive systems (Giacomuzzo et al., 2024), PACE proactively synchronizes assistance by continuously monitoring progress, aligning with human preferences for anticipatory support (Lasota and Shah, 2015). Subjective evaluations further confirmed that participants perceived PACE-driven robots as more intuitive partners, with improved fluency and responsiveness–a critical factor in fostering trust.

Our hybrid completion-time prediction method further validates the synergy between prior knowledge and real-time estimation. Its consistent performance across tasks demonstrates the practicality of combining historical data with online alignment, even in scenarios with inter-user variability. These results not only highlight the strengths of our approach but also underscore the broader potential of real-time progress estimation in transforming HRC systems.

Nonetheless, several limitations should be acknowledged. First, by relying on a single reference trajectory for OS-DTW_WP_, our method overlooks the fact that many actions admit multiple valid execution modes, which can differ substantially in movement patterns while still achieving the same goal. Moreover, our formulation exclusively leverages human motion, without incorporating additional state information–such as object pose or environmental cues–that could further improve action completeness estimation. Finally, the PACE framework assumes a fixed action sequence, whereas real-world collaborations often involve dynamic task orderings, execution errors, or mid-action changes in user strategies and preferences. Addressing these limitations will be essential for scaling our approach to more unstructured and unpredictable HRC settings.

### 5.1 Future works

Building on these findings, this work lays the foundation for several promising directions in human-robot interaction (HRI) and collaborative robotics. While validated in industrial assembly, our framework has the potential to generalize to diverse domains, such as home robotics, assistive care, and collaborative cooking. Additionally, we believe Dynamic Time Warping (DTW) can be extended to compute phase estimation accuracy and identify potential failures, paving the way for more robust and reliable systems.

A key area for future research is the integration of multi-modal sensing to enhance progress estimation. While our current framework relies on kinematic data, incorporating visual and contextual inputs–such as object affordances and environmental cues–could significantly improve flexibility and applicability. For instance, leveraging deep learning methods to process visual data could enable systems to handle unstructured tasks, such as improvised meal preparation or assistive caregiving, where task sequences and object interactions are highly variable. At the same time, because many tasks can be completed in multiple valid ways, future methods should be capable of modeling these multimodal distributions to anticipate diverse execution modes and adapt robot behavior accordingly.

Another critical direction is the development of frameworks that extend beyond rigid, predefined workflows. While PACE demonstrates robust performance in structured industrial tasks, real-world scenarios often involve unpredictable task sequences, errors, or mid-action changes in user preferences. Future systems must address these challenges by incorporating adaptable collaboration strategies, such as dynamic task re-planning and error recovery mechanisms. This would enable robots to seamlessly adjust to human actions, even in highly unstructured environments.

### 5.2 Conclusion

Beyond our technical contributions, this work highlights a broader imperative: real-time prediction of human action completion times remains vastly underexplored despite its critical role in seamless human-robot collaboration. While much of the existing research has overlooked action-level progress estimation, our results show that techniques like online DTW deliver significant improvements in synchronization and user satisfaction, as evidenced by the PACE framework. We aim to raise awareness within the HRC community about the urgent need for focused research in this area, and hope that this work inspires the development of new, effective learning methods. The efficiency gains, reduced idle times, and positive user feedback achieved with PACE illustrate the transformative potential of proactive, action-aware HRC systems—a paradigm shift essential for deploying robots in shared, real-world environments.

In conclusion, our results position real-time progress estimation as a cornerstone for collaborative robotics. By enabling robots to “keep pace” with humans at the level of individual actions, we unlock fluid, adaptive teamwork, where machines no longer wait for explicit cues but anticipate and align with human partners seamlessly. This shift from rigid synchronization to dynamic co-adaptation represents a critical leap toward truly intuitive human-robot collaboration.

## Data Availability

The raw data supporting the conclusions of this article will be made available by the authors, without undue reservation.

## Footnotes

1
https://www.ikea.com/us/en/p/ivar-chair-pine-90263902/

2
https://www.ros.org/

3
https://moveit.ai/

4
https://www.rokoko.com/products/smartgloves

5
https://haptx.com

6 Soft-DTW_EU_ was used to obtain the ground truths for the rail placing and screw placing tasks. However, this method was not effective for aligning one of the screw placing trajectories, in which case we employed Soft-DTW_WP_. Soft-DTW_WP_ was also used for the screwing task.
