# Supplementary material for: Real-time human progress estimation with online dynamic time warping for collaborative robotics
Source: Front Robot AI. 2025 Dec 4;12:1623884. doi: 10.3389/frobt.2025.1623884 (PMC12712710; doi:10.3389/frobt.2025.1623884)
Supplement: Supplementary file 1 [file Supplementaryfile1.pdf]

Supplementary Material

1 DYNAMIC TIME WARPING ALGORITHMS

For completeness, we report the Dynamic Time Warping (DTW) algorithm in Supplementary Algorithm 1, and of Soft-DTW in Supplementary Algorithm 2. Both algorithms are designed to handle multidimensional signals, accept arbitrary distance metrics, and return the phase.

For a more intuitive introduction to the Dynamic Time Warping algorithm, we recommend the online article *Time Series Similarity Using Dynamic Time Warping – Explained* by Mishra (2020)[1].

In Soft-DTW, the backward recursion involves an additional step. As described in the online notes *Machine Learning for Time Series – Notes from Lectures at ENSAI* by Tavenard (2021)[2], the matrix $\mathbf{E}$ represents the "soft-path" matrix that indicates, for each pair (i, j), how much it contributes to the overall alignment. Finally, an additional recursion is performed to obtain the optimal warping path.

[1] Mishra, A. (2020, December). *Time Series Similarity Using Dynamic Time Warping – Explained. Medium, Walmart Global Tech Blog*. Available at: https://medium.com/walmartglobaltech/time-series-similarity-using-dynamic-time-warping-explained-9d09119e48ec

[2] Tavenard, R. (2021). *Machine Learning for Time Series – Notes from Lectures at ENSAI*. Available at: https://rtavenar.github.io/ml4ts_ensai/contents/align/softdtw.html

Supplementary Algorithm 1 Dynamic Time Warping

Inputs:

- Query signal $\mathbf{a} = [\mathbf{a}_0, \dots, \mathbf{a}_{m-1}] \in \mathbb{R}^{m \times d}$
- Reference signal $\mathbf{b} = [\mathbf{b}_0, \dots, \mathbf{b}_{n-1}] \in \mathbb{R}^{n \times d}$
- Distance $\delta(\cdot, \cdot)$

Output:

- Phase $\boldsymbol{\tau} = [\tau_0, \dots, \tau_{m-1}] \in \mathbb{R}^m$ of $\mathbf{a}$ w.r.t. $\mathbf{b}$

Initialize $\mathbf{D} \in \mathbb{R}^{m \times n}$, where $D_{i,j} = \delta(\mathbf{a}_i, \mathbf{b}_j)$ ▷ Distance matrix computation

Initialize $\mathbf{R} \in \mathbb{R}^{(m+1)\times(n+1)}$, with $R_{0,0} = 0$, $R_{i,0} = \infty$ for $i \in [1, m]$, and $R_{0,j} = \infty$ for $j \in [1, n]$ ▷ Forward recursion

for $i = 1$ to m **do**
    for $j = 1$ to n **do**
        $R_{i,j} = D_{i-1,j-1} + \min(R_{i-1,j}, R_{i,j-1}, R_{i-1,j-1})$
    end for
end for

Set $i = m, j = n$ ▷ Backward recursion
while $i > 0$ or $j > 0$ **do**
    $\tau_{i-1} = j/(n-1)$
    if $i = 0$ **then**
        $j = j - 1$
    else if $j = 0$ **then**
        $i = i - 1$
    else
        Compute $\min(R_{i-1,j}, R_{i,j-1}, R_{i-1,j-1})$ and select the smallest one
        if $R_{i-1,j}$ is selected **then**
            $i = i - 1$
        else if $R_{i,j-1}$ is selected **then**
            $j = j - 1$
        else
            $i = i - 1$
            $j = j - 1$
        end if
    end if
end while

Supplementary Algorithm 2 Soft-DTW

Inputs:

- Query signal $\mathbf{a} = [\mathbf{a}_0, \ldots, \mathbf{a}_{m-1}] \in \mathbb{R}^{m \times d}$
- Reference signal $\mathbf{b} = [\mathbf{b}_0, \ldots, \mathbf{b}_{n-1}] \in \mathbb{R}^{n \times d}$
- Distance $\delta(\cdot, \cdot)$
- Smoothing parameter $\gamma \geq 0$

Output:

- Phase $\boldsymbol{\tau} = [\tau_0, \ldots, \tau_{m-1}] \in \mathbb{R}^m$ of $\mathbf{a}$ w.r.t. $\mathbf{b}$

Initialize $\mathbf{D} \in \mathbb{R}^{m \times n}$, where $D_{i,j} = \delta(\mathbf{a}_i, \mathbf{b}_j)$ ▷ Distance matrix computation
Initialize $\mathbf{R} \in \mathbb{R}^{(m+1)\times(n+1)}$, with $R_{0,0} = 0$, $R_{i,0} = \infty$ for $i \in [1, m]$, and $R_{0,j} = \infty$ for $j \in [1, n]$ ▷ Forward recursion
for $i = 1$ to m **do**
    for $j = 1$ to n **do**
        $R_{i,j} = D_{i-1,j-1} + \min^{\gamma}(R_{i-1,j}, R_{i,j-1}, R_{i-1,j-1})$
    end for
end for
Initialize $\mathbf{E} = \mathbf{O}_{(m+1)\times(n+1)}$, set $E_{m,n} = 1$ ▷ Backward recursion
Set $R_{m,n} = R_{m-1,n-1}$
Set $D_{m-1,n-1} = 0$
for $j = n - 1$ down to 1 **do**
    for $i = m - 1$ down to 1 **do**
        $a = \exp\left((R_{i+1,j} - R_{i,j} - D_{i,j-1})/\gamma\right)$
        $b = \exp\left((R_{i,j+1} - R_{i,j} - D_{i-1,j})/\gamma\right)$
        $c = \exp\left((R_{i+1,j+1} - R_{i,j} - D_{i,j})/\gamma\right)$
        $E_{i,j} = E_{i+1,j} \cdot a + E_{i,j+1} \cdot b + E_{i+1,j+1} \cdot c$
    end for
end for
Set $i = m, j = n$
while $i > 0$ or $j > 0$ **do**
    $\tau_{i-1} = j/(n-1)$
    if $i = 0$ **then**
        $j = j - 1$
    else if $j = 0$ **then**
        $i = i - 1$
    else
        Compute $\max(E_{i-1,j}, E_{i,j-1}, E_{i-1,j-1})$ and select the larger one
        if $E_{i-1,j}$ is selected **then**
            $i = i - 1$
        else if $E_{i,j-1}$ is selected **then**
            $j = j - 1$
        else
            $i = i - 1$
            $j = j - 1$
        end if
    end if
end while
